# Characterization of fluorescent probe substrates to develop an efficient high-throughput assay for neonatal hepatic CYP3A7 inhibition screening

**DOI:** 10.1038/s41598-021-98219-x

**Published:** 2021-09-30

**Authors:** Hannah M. Work, Sylvie E. Kandel, Jed N. Lampe

**Affiliations:** Department of Pharmaceutical Sciences, Skaggs School of Pharmacy and Pharmaceutical Sciences, University of Colorado Denver, Anschutz Medical Campus, Aurora, CO 80045 USA

**Keywords:** Screening, Oxidoreductases

## Abstract

CYP3A7 is a member of the cytochrome P450 (CYP) 3A enzyme sub-family that is expressed in the fetus and neonate. In addition to its role metabolizing retinoic acid and the endogenous steroid dehydroepiandrosterone sulfate (DHEA-S), it also has a critical function in drug metabolism and disposition during the first few weeks of life. Despite this, it is generally ignored in the preclinical testing of new drug candidates. This increases the risk for drug-drug interactions (DDI) and toxicities occurring in the neonate. Therefore, screening drug candidates for CYP3A7 inhibition is essential to identify chemical entities with potential toxicity risks for neonates. Currently, there is no efficient high-throughput screening (HTS) assay to assess CYP3A7 inhibition. Here, we report our testing of various fluorescent probes to assess CYP3A7 activity in a high-throughput manner. We determined that the fluorescent compound dibenzylfluorescein (DBF) is superior to other compounds in meeting the criteria considered for an efficient HTS assay. Furthermore, a preliminary screen of an HIV/HCV antiviral drug mini-library demonstrated the utility of DBF in a HTS assay system. We anticipate that this tool will be of great benefit in screening drugs that may be used in the neonatal population in the future.

## Introduction

Nationally, 6–8% of all newborns are admitted to the neonatal intensive care unit (NICU), and of those admitted, ~ 50–90% are prescribed off-label medications^1–6^. The high incidence rate of off-label drug use mainly stems from the lack of randomized clinical trials in this fragile patient population, leading to the use of adult dosing regimens that are adjusted solely for bodyweight and/or size differences^1,4,7^. Drug dosing in neonates is rarely based on neonatal physiology and, as a result, dosing regimens in neonates have been formed on a trial-and-error basis, at times resulting in either subtherapeutic doses or adverse drug reactions (ADRs) from overdosing^4,7,8^. For instance, Kaletra, a ritonavir-boosted lopinavir regimen used in newborns as a prophylactic treatment for HIV infection, has been reported to cause serious health problems, including renal toxicity and collapse, central nervous system impairment, cardiac arrhythmia, hemolysis and transient adrenal insuffiency^9–13^. Intriguingly, inhibition of the hepatic CYP3A7 enzyme by the Kaletra pharmacoenhancer ritonavir may be a leading cause of the substantial increase in plasma level of the adrenal steroid hormone DHEA-S and associated adrenal insufficiency observed in the treated newborns^9^.

A significant factor leading to ADRs like those experienced from Kaletra is the difference in the hepatic metabolism profile of neonates versus adults^9,14,15^. Newborns and fetuses predominantly express cytochrome P450 CYP3A7, which accounts for ~ 50% of the total CYP content in their livers, but express negligible amounts of CYP3A4/5^16^. CYP3A7 is important to the fetus and the neonate for proper growth and development, as it metabolizes endogenous substrates such as retinoic acid and DHEA-S^17,18^. Adults generally do not express this enzyme, but rather predominantly express CYP3A4, which accounts for 10–50% of the CYP content in their liver and metabolizes roughly 50% of marketed drugs^17,18^. Interestingly, the expression of CYP3A7 is highest just before birth and gradually decreases during the first few months of life while CYP3A4 expression increases concomitantly, presenting a unique isoform switching phenomenon occurring with these closely related enzymes^16,18^. While CYP3A4 and CYP3A7 are almost 88% identical in amino acid sequence and share similar substrate profiles, these enzymes metabolize drugs at significantly different rates. Previous work has demonstrated that CYP3A7 has a substantially lower k_cat_ compared to CYP3A4, typically greater than tenfold lower for most drug substrates, making CYP3A7 significantly less catalytically active^17,19–21^. Despite CYP3A7’s lower catalytic activity and importance in the neonatal population, this enzyme is not well-studied nor typically investigated pre-clinically when estimating the safety and efficacy profile of new drug candidates, primarily due to its similarity to CYP3A4 and assumed identical metabolic activity. Considering the differing CYP3A enzyme activity of neonates versus adults, neonates are at much higher risk of experiencing ADRs from drug-drug interactions (DDI) if CYP3A7 is inhibited by an administered medication. To help identify the risk new drugs pose to neonates and developing infants prior to administration, a reliable model must be developed that can accurately detect CYP3A7 inhibitors, preferably in a high-throughput screening fashion for industrial purposes.

Currently, initial safety estimations are evaluated by studying the metabolic activity of the major adult enzymes in the CYP family^22^. Drugs that are strong CYP inhibitors or those that are not cleared well metabolically have a decreased likelihood of reaching human clinical trials and eventually becoming an approved drug due to the high probability of DDI or other toxic liabilities. Metabolism is initially assessed by screening potential new drug candidates using in vitro systems containing heterologously expressed recombinant human CYP enzymes and/or human liver microsomes (HLMs)^22^. Given that the supply of neonatal HLMs is limited due to the low incidence of neonatal mortality in the US, recombinant CYP3A7 is typically employed to assess drug clearance by the CYP3A pathway in neonates and developing infants. These in vitro systems normally rely on a marker substrate for enzyme activity, such as a fluorescent or luminescent probe. Such probes exist commercially for CYP1A2, 2B6, 2C9, 2C19, 2D6, 3A4, and 3A5^23^, but none have been identified specifically for CYP3A7. Recently, luminescent probes originally developed as CYP3A4 substrates have also been marketed for CYP3A7^24–27^. One disadvantage with these type of probes in HTS assays is that the bioluminescence is produced indirectly via the activity of a secondary enzyme, luciferase in this case. Because of this, these types of assays are more sensitive to negative interference through direct inhibition of the luciferase enzyme. This can generate a high number of false-positive inhibitors, as drug compounds may inhibit the luciferase enzyme rather than the CYP^28^. Additionally, luciferin-based assays are much more expensive than most standard fluorescence assays, making them less enticing for industrial use.

The present study aimed to identify a commercially available fluorophore that can assess CYP3A7 activity and inhibition in a high-throughput screening format. We tested a broad array of fluorescent probes that varied in molecular weight, solubility, and fluorescence parameters (Table 1). All probe substrates undergo *O*-dealkylation reactions to produce their fluorescent product, except for Nile Red, which undergoes sequential *N*-dealkylation reactions^29^. Amount of product formed and its relationship with respect to time were determined for each probe substrate. Timepoints where the rate of product formation was linear with respect to time were used as end points for the individual incubations to ensure steady-state conditions. The maximal rate of reaction (*V*_max_), Michaelis–Menten constant (*K*_M_), signal-to-noise (S/N) ratio, Z′ factor, and cost per 96-well microtiter plate were evaluated for each fluorophore. We determined that the fluorophore dibenzylfluorescein (DBF) was superior in its performance when compared to other compounds in meeting all the criteria considered for an efficient HTS assay. DBF was then tested by screening a mini-library of HIV/HCV antiviral drugs to identify known and unknown CYP3A7 inhibitors. Furthermore, in order to validate DBF as a suitable CYP3A7 probe substrate for the HTS inhibition assay, the IC_50_ values for a known endogenous CYP3A7 substrate, DHEA-S, and two known inhibitors, ritonavir and lopinavir, were determined.

**Table 1:**
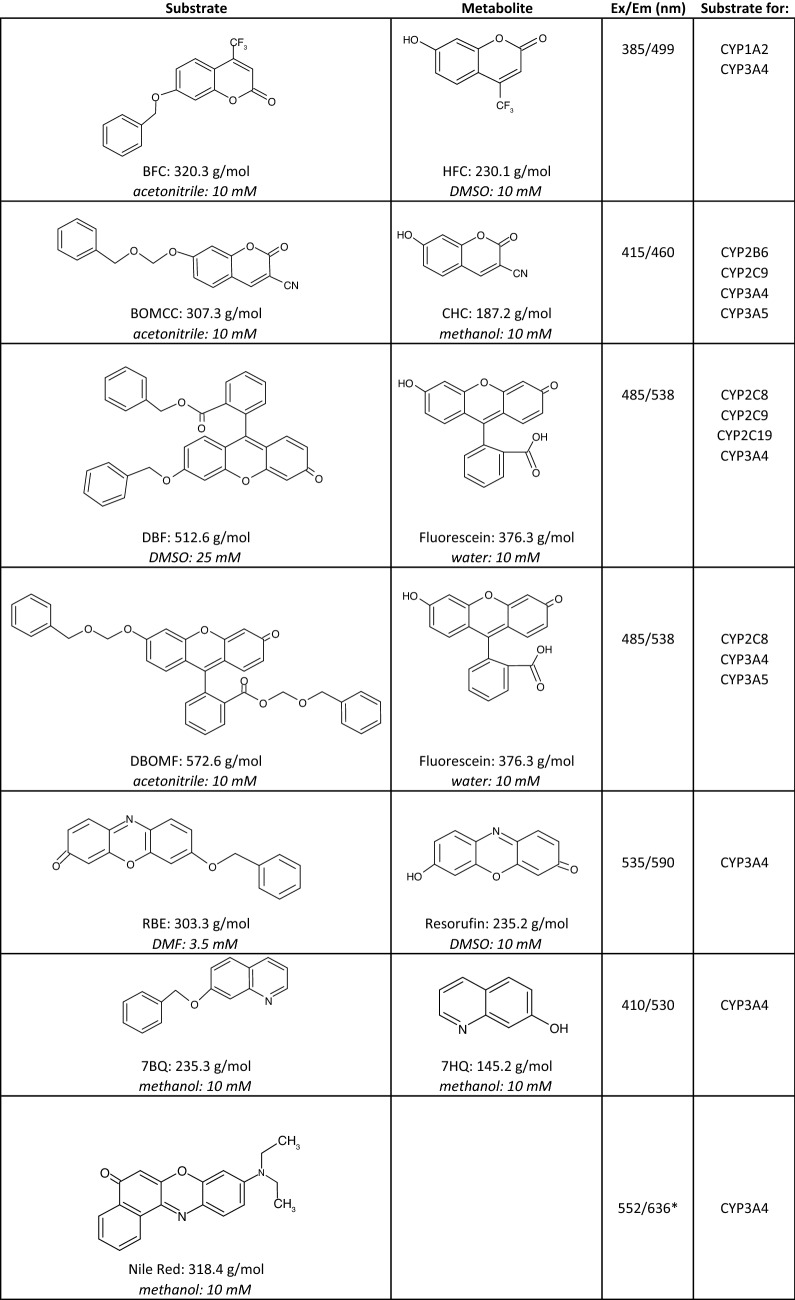
Fluorogenic substrates, their respective metabolite excitation and emission wavelengths, solubility constraints, and reported CYP P450 selectivity.

BFC, 7-benzyloxy-4-trifluoromethyl-coumarin; BOMCC, 7-benzyloxymethyloxy-3-cyano-coumarin; CHC, 3-cyano-7-hydroxycoumarin; DBF, dibenzylfluorescein; DBOMF, dibenzyloxymethylfluorescein; HFC, 7-hydroxy-4-trifluoromethyl-coumarin; RBE, resorufin benzyl ether; 7BQ, 7-benzyloxy-quinoline; 7HQ, 7-hydroxyquinoline.

*Excitation and emission wavelengths are representative of the parent compound.

## Materials and methods

### Chemicals and enzymes

Insect cell microsomes prepared from baculovirus-infected Sf9 cells expressing human CYP3A7 enzyme (Supersomes, catalog # 456237) were purchased from Corning (Corning, NY). These Supersomes contained recombinant CYP3A7 enzyme co-expressed with human NADPH-cytochrome P450 reductase and human cytochrome *b*_5_. The probe substrates and their respective fluorescent metabolites are presented in Table 1. Fluorescein sodium salt (FLU, CAS: 518-47-8), 3-cyano-7-hydroxycoumarin (CHC, CAS: 19088-73-4), 7-hydroxy-4-trifluoromethyl-coumarin (HFC, CAS: 575-03-01), Nile Red (NR, CAS: 7385-67-3), resorufin benzyl ether (RBE, CAS: 87687-02-3), glucose-6-phosphate, glucose-6-phosphate dehydrogenase, *β*-nicotinamide adenine dinucleotide phosphate (NADP^+^), and dehydroepiandrosterone sulfate (DHEA-S, CAS: 651-48-9) were all obtained from Sigma Aldrich (St. Louis, MO). 7-hydroxyquinoline (7-HQ, CAS: 580-20-1), ketoconazole (CAS: 65277-42-1), 7-benzyloxymethyloxy-3-cyano-coumarin (BOMCC, catalog # P2980), and dibenzyloxy-methylfluorescein (DBOMF, catalog # P2979) were obtained from Thermo Fisher Scientific (Waltham, MA). Dibenzylfluorescein (DBF, CAS: 97744-44-0), resorufin sodium salt (RSS, CAS: 34994-50-8), lopinavir (CAS: 192725-17-0), and ritonavir (CAS: 155213-67-5) were obtained from Cayman Chemical (Ann Arbor, MI). 7-Benzyloxyquinoline (7-BQ, catalog # CYP512) was purchased from Cypex (Dundee, Scotland, UK), and 7-benzyloxy-4-trifluoromethylcoumarin (BFC, catalog # 451730) was purchased from Corning (Corning, NY). HIV/HCV antiviral inhibitors were from an antiviral inhibitor library obtained from Selleck Chemicals (Houston, TX) (Table 2), all of which were pre-dissolved in dimethylsulfoxide (DMSO) to a concentration of 10 mM. All other chemicals and solvents used were obtained from standard suppliers and were of reagent or analytical grade.

**Table 2:** List of tested inhibitors, drug class, and reported effects on CYP3A7.

Compound	MW (g/mol)	Drug class	Known CYP3A7 effects
Boceprevir	519.7	HCV protease inhibitor	N/A
Danoprevir	731.8	HCV protease inhibitor	N/A
Dasabuvir	493.6	HCV NNRTI	N/A
Glecaprevir	838.9	HCV protease inhibitor	N/A
Grazoprevir	766.9	HCV protease inhibitor	N/A
Ledipasvir	889	HCV phosphoprotein inhibitor	N/A
Lomibuvir	445.6	HCV NNRTI	N/A
Ombitasvir	894.1	HCV phosphoprotein inhibitor	N/A
Simeprevir	749.9	HCV protease inhibitor	N/A
Telaprevir	679.9	HCV protease inhibitor	N/A
Amprenavir	505.6	HIV protease inhibitor	Inhibitor
Ataznavir	704.9	HIV protease inhibitor	N/A
Darunavir	547.7	HIV protease inhibitor	N/A
Lopinavir	628.8	HIV protease inhibitor	Inhibitor
Nelfinavir	663.9	HIV protease inhibitor	Inhibitor
Ritonavir	720.9	HIV protease inhibitor, pharmacoenhancer	Inhibitor

NNRTI: non-nucleoside reverse transcriptase inhibitor.

### Chemical stock and working solutions

DBF, HFC, RSS, and ketoconazole were dissolved in DMSO (10–25 mM stock); BFC, BOMCC, and DBOMF were dissolved in acetonitrile (ACN) (10 mM stock); CHC, 7-BQ, Nile Red, and 7-HQ were dissolved in methanol (10 mM stock); RBE was dissolved in dimethylformamide (DMF) (3.5 mM stock); and fluorescein was dissolved in water (10 mM stock). All probe substrates were serially diluted twofold in their respective solvents to prepare working solutions with concentrations ranging between 0.01 to 2.5 mM. The fluorescent metabolites were diluted in their respective solvent to produce metabolite standard calibration curves ranging from 0.20 to 100 µM. DHEA-S, lopinavir, and ritonavir were dissolved in DMSO to a concentration of 50 mM, 40 mM, and 10 mM, respectively, then diluted to obtain working solutions ranging between 2.44 nM to 50 mM.

### Fluorogenic substrate incubations with recombinant CYP3A7 enzyme

Assays were conducted in triplicate in 96-well black polystyrene microtiter plates (Costar, catalog # 266) in a 100 µL volume and prepared on ice. The reactions contained 10 pmol/mL of CYP3A7 Supersomes, 0.1 M potassium phosphate buffer (pH 7.4), and 3.3 mM magnesium chloride. Two probe substrate concentrations between 3.5 and 50 µM were tested in order to determine the initial linearity of product formation. DMSO and DMF concentrations were kept below 0.2% of the final reaction volume, and methanol and acetonitrile concentrations were kept below 0.5% to minimize non-specific CYP inhibition. Enzyme/substrate (E/S) mixes (80 µL) were preequilibrated at 37 °C for 3–5 min and reactions were initiated by the addition of the NADPH-regenerating system mix (20 µL) (at final concentrations in the incubations of 1 mM NADP^+^, 10 mM glucose-6-phosphate and 2 IU/mL of glucose-6-phosphate dehydrogenase). Fluorescent signal was measured using the Tecan Infinite 200 PRO plate reader at the corresponding metabolite λ_max_ fluorescence emission wavelength, as listed in Table 1. For substrates BFC, BOMCC, RBE, Nile Red, and 7-BQ, fluorescent signal was measured directly every 2.5 min for 90 min. For substrates DBF and DBOMF, reactions were terminated at timepoints between 0 and 60 min by the addition of 75 µL 2 M sodium hydroxide (NaOH) or 50 µL 0.5 M Tris base, respectively. For the 0 min timepoint, the stop buffer was added first, followed by the NADPH-regenerating mixture (“stop-before-start”). After adding the stop buffer, the plate was incubated at 37 °C for an additional hour in order to liberate the fluorescein fluorophore. Metabolites were quantified based on calibration curves prepared in buffer mix without the recombinant CYP3A7 present in order to account for any matrix effects. Metabolite formation was evaluated for linearity by fitting the data to a linear regression equation using GraphPad Prism (version 8.2.1, GraphPad Software, La Jolla, CA). Reactions with (1) no CYP3A7, (2) no NADPH-regenerating system mix, and/or (3) NADPH-regenerating system mix addition after stopping the reaction were used as negative controls.

### Kinetic constant determination

Assays were conducted in triplicate in 96-well black microtiter plates in a 100 µL volume. Reactions were prepared as described above with substrate concentrations ranging between 0.02 and 50 µM and terminated by the addition of 75 µL 80:20 acetonitrile:0.5 M Tris base for 7-BQ, BFC, and RBE after 50, 60, and 90 min, respectively. DBF and DBOMF reactions were stopped as described above after 45 min and 10 min, respectively, then incubated for an additional hour at 37 °C in the presence of strong base in order to liberate the fluorescein fluorophore. BOMCC reactions were terminated by the addition of 50 µL 0.5 M Tris base after 55 min. Fluorescent signal was measured using the Tecan Infinite 200 PRO plate reader, and metabolites were quantified using corresponding metabolite standard calibration curves. The velocity was calculated by applying Eq. (1):

1 $$v=\frac{[m]}{t\cdot [E]}$$

where *[m]* is the concentration of metabolite produced (pmol/L), *t* is time (min), and *[E]* is the enzyme concentration (pmol/L). The Michaelis–Menten constant (*K*_M_) and the maximal rate of reaction (*V*_max_) values were assessed via GraphPad Prism software (version 8.2.1, GraphPad Software, La Jolla, CA) by fitting the mean metabolite formation rate data (n = 3) to the Michaelis–Menten equation.

### Assessment of assay quality

Assays were conducted in 96-well black microtiter plates as described above. Substrate concentrations slightly below their respective *K*_M_ values were used. A total of 12 replicates were performed and the fluorescent signal was measured. Assay quality was assessed by calculating the signal-to-noise (S/N) ratio (Eq. 2) and Z′ factor (Eq. 3) for each substrate, where *µ* and *σ* represent the mean and standard deviation of the assay signal, respectively, of the positive (+) and negative (−) controls. Reactions containing no CYP3A7 were used as negative controls for purposes of metabolite quantification.

2 $$S/N = \frac{{\mu }_{+}-{\mu }_{-}}{{\sigma }_{-}}$$

3 $${Z}^{^{\prime}}factor=1-\frac{3{\sigma }_{+}+3{\sigma }_{-}}{|{\mu }_{+}-{\mu }_{-}|}$$

### CYP3A7 inhibition screening of an HIV/HCV inhibitor mini-library

DBF and 7-BQ were further investigated for their potential to assess the inhibitory effects of antiviral inhibitors against CYP3A7. Assays were conducted in triplicate as described above and contained either 0.156 µM DBF or 0.375 µM 7-BQ, concentrations being below the *K*_M_ of each substrate. HIV/HCV inhibitors were aliquoted to the E/S mixes to a final concentration of 20 µM. The fluorescent signal was measured using the Tecan Infinite 200 PRO plate reader. Reactions containing (1) no CYP3A7, (2) solvent control, (3) ketoconazole, and (4) NADPH-regenerating system mix addition after stopping the reaction were used as controls. Percent inhibition was calculated using Eq. (4), where *RFU* represents the relative fluorescence units, *test* refers to the signal from reactions containing a HIV/HCV inhibitor, and (-) represents the signal from the solvent control reactions. Reactions containing no CYP3A7 were used as the *background* control.

4 $$\%\, inhibition=\frac{{{RFU}_{\left(-\right)}-RFU}_{test}-{RFU}_{background}}{{RFU}_{(-)}-{RFU}_{background}}$$

### IC_50_ assays for recombinant CYP3A7 enzyme using DBF as probe substrate

DHEA-S, lopinavir, and ritonavir IC_50_ assays were conducted in triplicate as described above and each contained 0.156 µM DBF as the primary substrate. In order to obtain an accurate IC_50_ value, inhibitor concentrations were varied and tested across a concentration range > 2 orders of magnitude. Inhibitor compounds were aliquoted to E/S mixes to final concentrations ranging between 4.88 nM and 100 µM while keeping the DMSO concentration below 0.2% of the final volume. Reactions containing (1) no CYP3A7, (2) solvent control, (3) ketoconazole, and (4) NADPH-regenerating system mix addition after stopping the reaction were used as controls. Percent inhibition was calculated as described above, and data were fitted to a nonlinear regression model ([Inhibitor] vs. response (three parameters)) in GraphPad Prism (version 9.1.1), considering only the mean Y value of each point.

### Cost analysis

The cost of CYP3A7 Supersomes, all components of the NADPH-regenerating mixture, E/S mix buffers, along with the fluorophore probes and their respective metabolites, solvents, and stop buffers were all incorporated into the final cost to run 96 reactions (one full plate). The cost of supplies to generate metabolite standard curves was also factored into the total cost. Analyses were performed in Excel (Microsoft Software, Redmond, WA).

## Results

### Linearity and kinetic assessment of recombinant CYP3A7 metabolic activity for different fluorescent probes

**Figure 1:**
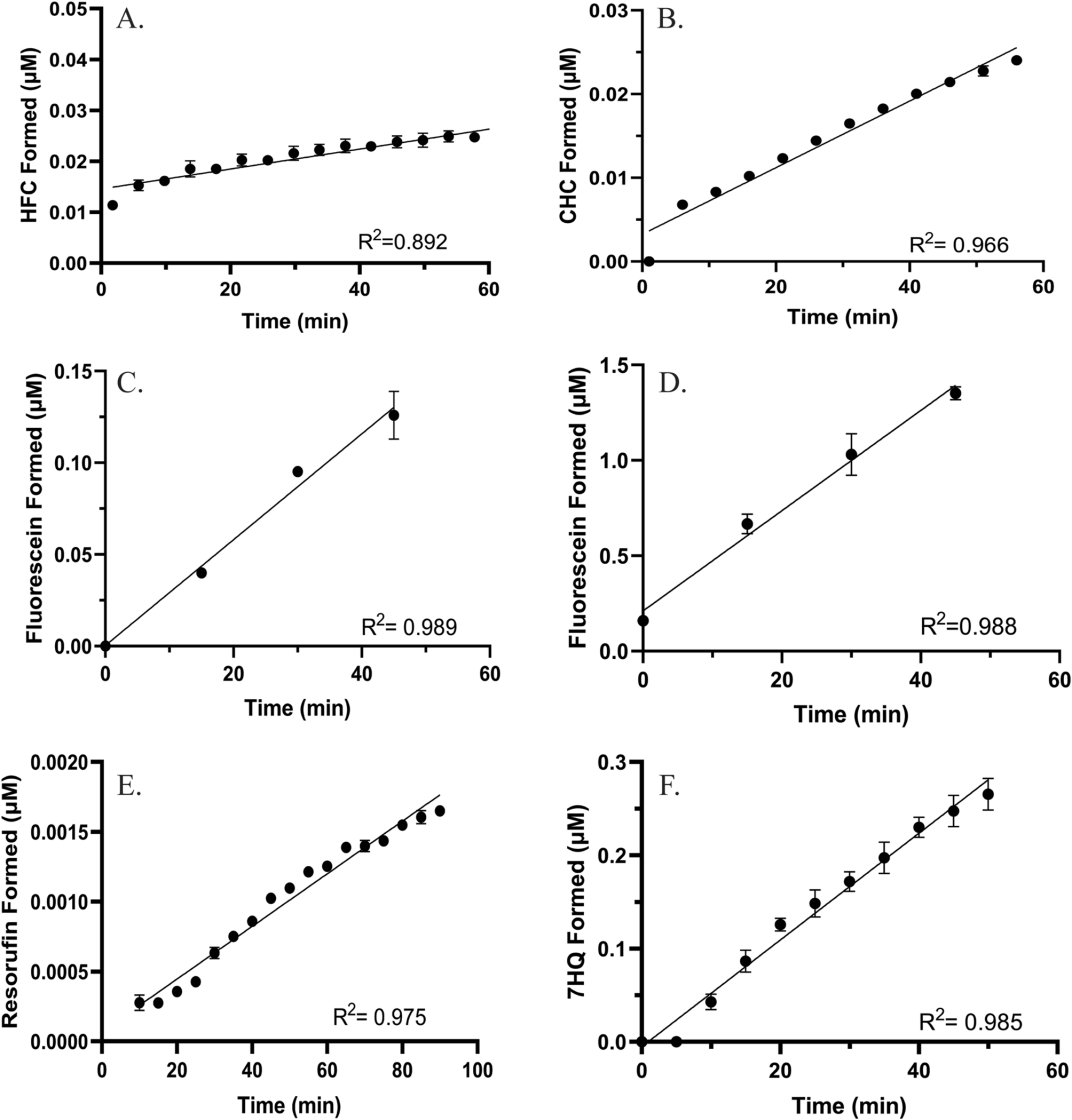
Time dependence of the initial velocity (V0) of (**A**) BFC; (**B**) BOMCC; (**C**) DBF; (**D**) DBOMF; (**E**) RBE; and (**F**) 7BQ at 5 µM. Each point represents the average of 3 replicates, and the error bars represent the standard deviation. All data was fit to a linear regression line.

The incubation time for each assay was established by exploring the linearity of metabolite formation in each reaction conducted with the recombinant CYP3A7 (Fig. 1). For drug-screening applications, it is essential that reactions are run under steady state conditions and that less than 10% of substrate depletion occurs to ensure that the substrate concentration does not change significantly. Metabolite formation was measured by fluorescence for all substrates tested, except for Nile Red (Supplemental Figure S1). Previous reports demonstrate that Nile Red and its metabolites have similar and overlapping excitation and emission wavelengths^29,30^; thus, signal from the metabolite in the presence of the parent cannot be deciphered by fluorescence alone. Some substrate reactions (BFC, DBOMF, and RBE) do not start at 0 µM formation due to high background fluorescence from the substrate parent; the background is significantly reduced when using lower substrate concentrations. The reaction linearity varied between substrates, however all reactions remained under 10% of substrate depletion during the time examined except for DBOMF. BFC, BOMCC, and RBE were all poorly metabolized by recombinant CYP3A7, producing less than 0.6% of their respective metabolites. Metabolite formation was linear up to 45 min for DBF and DBOMF, up to 55 min for BOMCC, up to 60 min for 7-BQ and BFC and up to 90 min for RBE. Linearity was not investigated past 90 min due to concerns of CYP3A7 instability and loss of enzyme activity. Linearity of the metabolite formation for each probe was used as a guideline to conduct Michaelis–Menten kinetic assays, as well as drug screening inhibition and IC_50_ assays, as described in the Materials and Methods section.

**Figure 2:**
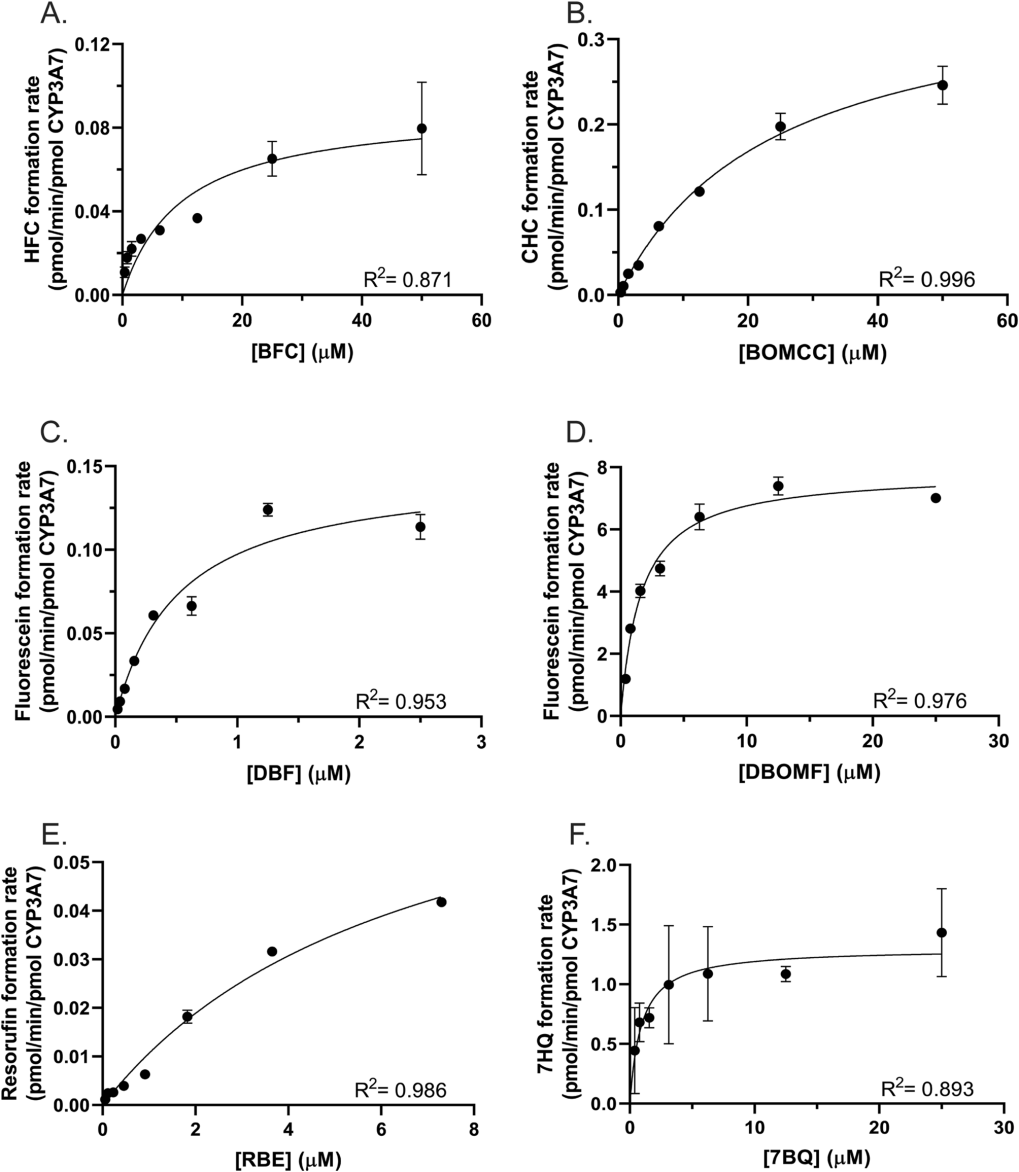
CYP3A7 Michaelis–Menten kinetics of (**A**) BFC; (**B**) BOMCC; (**C**) DBF; (**D**) DBOMF; (**E**) RBE; and (**F**) 7BQ. Each point represents the mean of triplicate measurements, with error bars representing standard deviations. Data were fitted to the hyperbolic non-linear regression line of the Michael-Menten model in GraphPad Prism.

Michaelis–Menten kinetic assays were used to determine the maximal rates of reaction (*V*_max_) and the Michaelis–Menten constants (*K*_M_) for each probe substrate examined. *K*_M_ and *V*_max_ values for each fluorogenic substrate were determined using specific concentration ranges for each substrate. The metabolite formation rate data were fitted to the hyperbolic Michaelis–Menten model (Fig. 2). The apparent *K*_M_ and *V*_max_ values varied significantly between the substrates tested (Table 3). DBF exhibited the highest affinity for CYP3A7 (0.52 µM), but a low turnover rate (0.15 pmol product/min/pmol CYP3A7) compared to the other substrates. DBOMF displayed the highest turnover rate (7.85 pmol product/min/pmol CYP3A7) and a relatively moderate affinity for CYP3A7 (1.62 µM) compared to the other substrates tested. 7-BQ exhibited both a moderate affinity (0.91 µM) and turnover rate (1.30 pmol product/min/pmol CYP3A7). All other substrates displayed both a lower affinity and turnover rate, which is consistent with the low product formation for each, as seen in Fig. 1.

**Table 3:** Comparison of fluorescent substrates’ kinetics, assay time, quality, and cost.

Fluorescent substrate	BFC	BOMCC	DBF	DBOMF	RBE	7BQ
Apparent *Km* (µM) (95% CI)	9.96 (2.27–44.8)	23.9 (18.5–31.4)	0.525 (0.247–1.16)	1.62 (1.06–2.44)	6.74 (3.74–14.9)	0.913 (0.379–1.97)
Apparent *Vmax* (pmol/min/pmol CYP3A7) (95% CI)	0.0896 (0.0578–0.172)	0.369 (0.328–0.423)	0.148 (0.114–0.204)	7.85 (7.02–8.79)	0.0825 (0.0597–0.139)	1.30 (1.08–1.56)
Total assay time (min)	60	55	105	70	90	50
Relative cost per plate ($)	72.58	125.81	70.96	78.84	75.35	71.15
S.N. ratio	87.4	349	222	381	96.4	41.9
Z′ factor	0.845	0.918	0.889	0.874	0.746	0.726

### Assay quality and cost assessment

To evaluate the reliability of the CYP3A7 HTS assay, Z′ factors and S/N ratios were determined. The Z′ factor is a statistical analysis parameter that reflects the performance of a screening platform. Assay platforms with Z′ values between 0.5 and 1.0 are considered robust and reliable, and S/N ratios above 10.0 are preferred. Reactions with and without the CYP3A7 enzyme were conducted, and the fluorescent output was measured and used to determine the Z′ factors and S/N ratios. The results are summarized in Table 3. BOMCC was observed to have the greatest S/N ratio and Z-score of all substrates investigated. Overall, all substrates met acceptable requirements to be considered robust and reliable.

To compare the cost-efficiency of this CYP3A7 inhibition HTS assay for the different probe substrates, the total cost to screen one full 96-well microtiter plate was calculated for each probe (Table 3). The total cost calculations included the cost of buffers, solvents, substrate, CYP3A7 Supersomes, NADPH regeneration system mix, and materials needed to prepare the metabolite standard curve. The overall cost did not vary significantly between substrates and ranged between $70 and $80 per plate, except for BOMCC, which was over $125. The CYP3A7 inhibition HTS assay utilizing DBF was the least expensive ($70.96).

### Reliability of the CYP3A7 in vitro HTS assay for drug inhibition assessment

**Figure 3:**
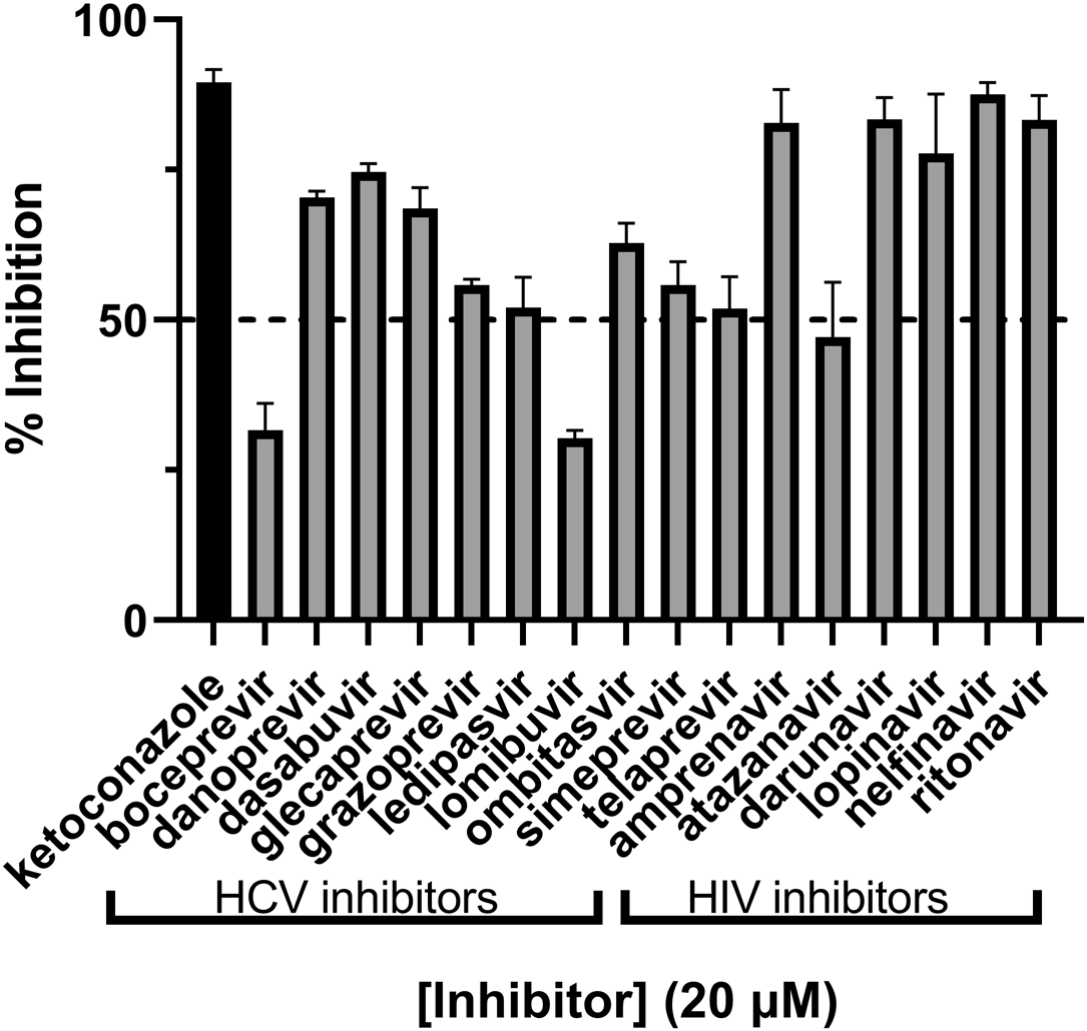
CYP3A7 inhibition by HCV and HIV inhibitors assessed using DBF as a fluorescent marker. All compounds were tested in triplicate. Bars represent the mean inhibition value, and the error bars represent the standard deviation.

DBF and 7-BQ exhibited greater affinities for CYP3A7, low assay cost, and above acceptable assay qualities compared to other substrates (Table 3). These two probes were then used to assess the inhibitory effects of drug compounds (at one concentration of 20 µM) against CYP3A7. Drug screening using 7-BQ as a marker for activity was unsuccessful due to the high variability in the assay and fluorescence background interference (Supplemental Figure S2). However, drug screening with the DBF substrate was conclusive (Fig. 3). Of the 16 HIV/HCV inhibitors tested at the concentration of 20 µM, 13 inhibited CYP3A7 activity by more than 50%. The inhibitor used as positive control, ketoconazole, showed 90% inhibition. Amprenavir, darunavir, lopinavir, nelfinavir and ritonavir showed the highest percent inhibition (> 75%) between all HIV/HCV inhibitors tested.

### DBF as a valid probe for CYP3A7 inhibition by DHEA-S, ritonavir, and lopinavir

An ancillary assessment of DBF metabolism by CYP3A7 confirmed that DBF underwent similar metabolism to previously characterized CYP enzymes^31^ (Supplemental Figure S3). Here, DBF metabolites formed in CYP3A7 reactions were further characterized by liquid chromatography tandem mass spectrometry (LC–MS/MS) (Supplemental Figure S4; Supplemental Table S1). Indeed, we observed that *O*-debenzylation of DBF was the major CYP3A7-related activity (Supplemental Figure S4, Panel A) and fluorescein was detected at higher abundance after alkaline treatment (Supplemental Figure S4, Panel B). However, in order to further validate DBF as a reliable probe for assessing CYP3A7 inhibition, the half-maximal inhibitory concentrations (IC_50_) of both a known CYP3A7 endogenous steroid substrate, DHEA-S, and two known CYP3A7 inhibitors; the HIV protease drugs ritonavir and lopinavir, were determined.

**Figure 4:**
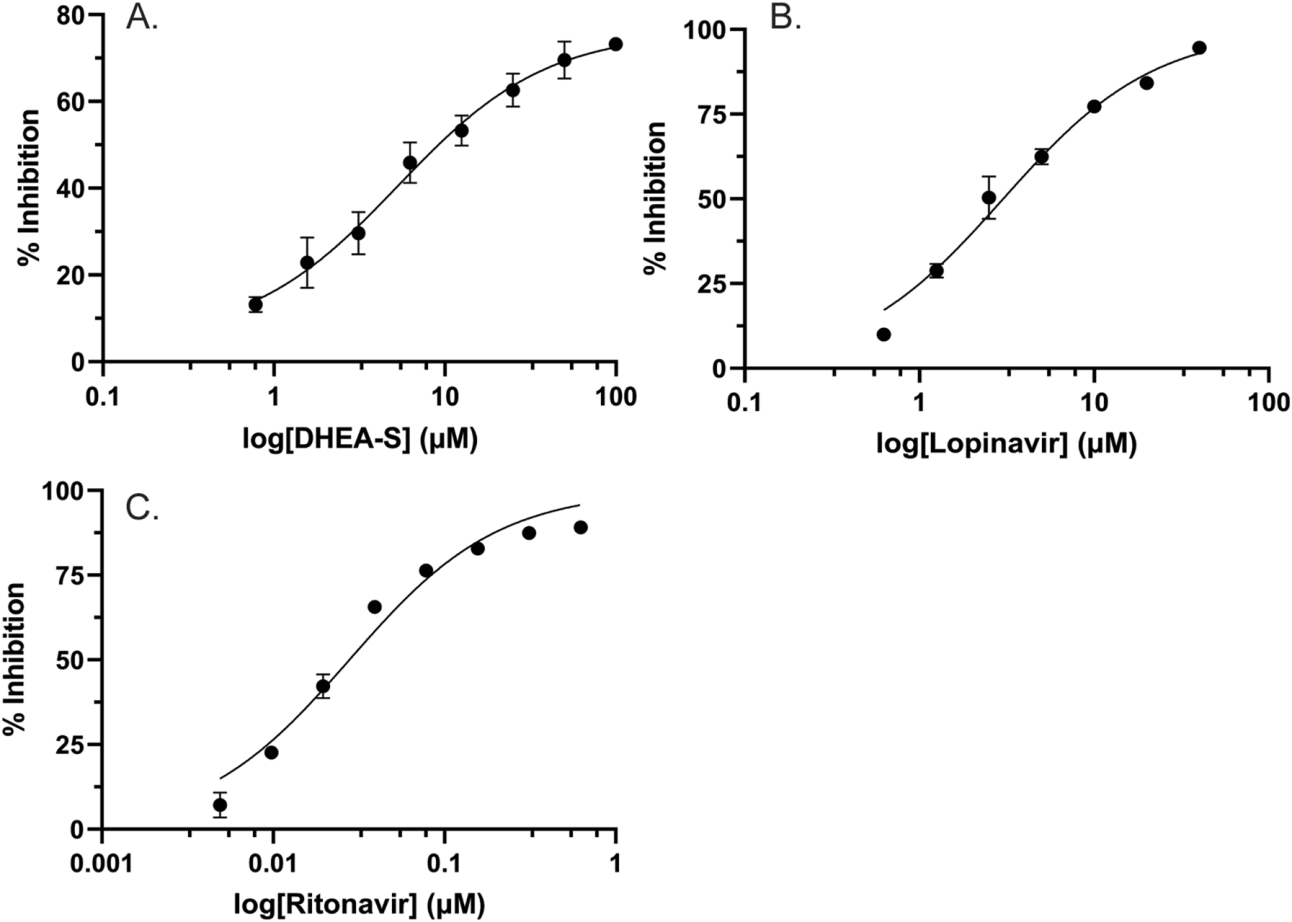
CYP3A7 IC50 inhibition curve by (**A**) DHEA-S, (**B**) Lopinavir, and (**C**) Ritonavir assessed using DBF as a fluorescent marker. All concentrations were tested in triplicate. Points represent the mean values, and error bars represent the standard deviations. Data were fitted to the non-linear regression [inhibitor] vs. response model in GraphPad Prism, and the best-fit value was (**A**) 5.31 µM, (**B**) 3.01 µM, and (**C**) 0.0278 µM.

For DHEA-S, previous work reports the *K*_M_ value of 16α-hydroxylation via CYP3A7 to be 5.4 µM^9^. The DHEA-S IC_50_ value against CYP3A7 metabolism of DBF we obtained was 5.31 µM, corresponding closely to the *K*_M_ for DHEA-S, assuming a strictly competitive model of inhibition (Fig. 4A). The IC_50_ obtained for lopinavir in the DBF assay with CYP3A7 was 3.01 μM (Fig. 4B), whereas the IC_50_ for ritonavir was 0.0278 μM (Fig. 4C). In comparison, the values reported previously in Kandel et al.^9^ were 5.88 μM for lopinavir and 0.0514 μM for ritonavir.

## Discussion

The development of sensitive, low-cost, and quick fluorometric assays for hepatic drug metabolizing CYP enzymes has enhanced our ability to assess the potential of new drug candidates and other xenobiotics for CYP inhibition that may pose a risk for DDI and toxicity. It is common industrial practice to screen drugs against major hepatic CYP enzymes that are expressed in adults, but it is not routine or standard to test for the inhibitory effects of these drugs on CYP3A7, despite the continued off label use of drugs in the neonatal patient population. The lack of preclinical testing of drugs for CYP3A7 inhibition in a neonate-relevant model has led to downstream adverse drug reactions and toxic effects in clinical settings^9,19,32–35^. Adopting and applying a standard, simple assay to screen for CYP3A7 inhibition would significantly reduce the possibility of CYP3A7-mediated adverse drug reactions by identifying potent CYP3A7 inhibitors prior to drug administration, thereby allowing the clinician to select a safer alternative.

**Figure 5:**
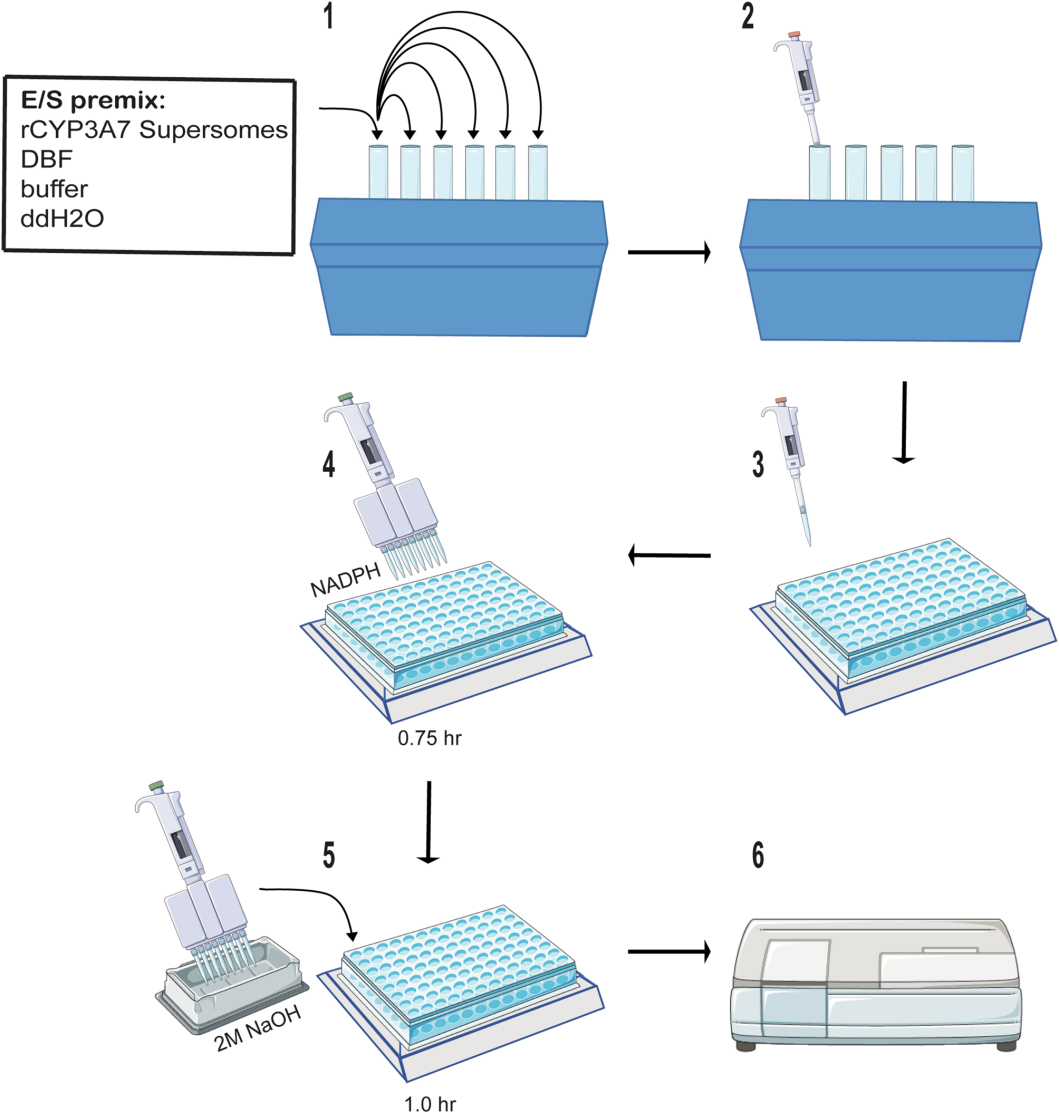
High-throughput screening fluorescent assay flowchart. (1) Prepare E/S premix on ice and distribute to glass vials. (2) Add test compound to respective glass vial. (3) Transfer 80 µL of E/S mixes to corresponding wells on plate. Pre-incubate plate at 37 °C for 3 min. (4) Start reactions by adding 20 µL of NADPH regenerating system mix; shake and incubate reaction for 45 min. (5) Add 75 µL of 2 M NaOH to each reaction; continue incubating for an additional hour. (6) Measure fluorescence of metabolite at Ex/Em wavelength of 485/538 nm using a fluorescence plate reader.

To this end, we sought to identify a fluorescent substrate for CYP3A7 that would exhibit the desired properties of cost, speed, S/N ratio, and Z′-factor (Table 3). While the use of luciferin-based assays has increased in recent years^24–27^, these were not considered here because of concerns of luciferase inhibition by the test article that could confound interpretation of the assay results. Additionally, these assays did not meet our criteria of cost and speed when compared with the fluorescent substrates. Given the high degree of substrate overlap between CYP3A4/5 and CYP3A7, we initially selected fluorescent compounds that were known CYP3A4/5^36–39^ and in some cases CYP3A7^37^, substrates and, from there, eliminated compounds based on cost, solubility constraints, or low enzyme affinity (*K*_M_). This reduced the number of final compounds to be tested to the seven shown in Table 1. Of these seven, Nile red was eliminated due to the lack of commercially available metabolites and overlap of the metabolite fluorescence emission profile with the fluorescence emission of the parent compound^29,30^. While all six remaining compounds tested were metabolized to a greater or lesser extent by CYP3A7 (Fig. 2, Table 3), dibenzylfluorescein (DBF) was found to be a superior substrate in respect to the parameters considered. The fluorescence signal is produced by CYP3A7 *O*-dealkylation of the benzyl ether moiety followed liberation of the fluorescein fluorophore by base-catalyzed ester hydrolysis (Supplemental Figure S3)^31^. *O*-dealkylation is a common and energetically facile reaction for CYP enzymes and generally leads to high product yields^40^. LC–MS/MS experiments conducted on the metabolites of the reaction confirmed that CYP3A7 did indeed perform the benzyl ether *O*-dealkylation of DBF (Supplemental Figure S4). DBF possesses a high affinity towards CYP3A7 (*K*_M_ value ~ 0.5 µM) and exhibits an above-acceptable assay quality, with a Z′-score of 0.89 and a signal-to-noise (S/N) ratio of 221.6 (Table 3), as well as a low assay cost ($70.96/96-well plate). While the DBF assay is slightly more time-consuming than some of the other substrate assays examined in this study due to its additional incubation period after stopping the reaction, it was found that this time can be maximized by preparing successive screening plates during the secondary incubation (Fig. 5). The use of automated robotic machinery, a common practice in industrial settings, may also speed the process to prepare plates, start/stop reactions, and screen potential drug candidates using this methodology. Additionally, DBF was less susceptible to allosteric activation whereby the addition of a second, chemically unrelated, xenobiotic substrate activates the metabolism of the first^41–43^, whereas BFC, RBE, 7-BQ (Fig. 2), and Nile red (not shown) all have previously been reported to display allosteric kinetics^29,37^. This type of kinetic phenomenon can convolute data interpretation and lead to spurious conclusions regarding the metabolism of a particular substrate or inhibitor^43^. Therefore, eliminating this effect as an assay variable is highly desirable.

To further validate the DBF assay, we determined the IC_50_ value of the steroid DHEA-S, a known endogenous substrate for CYP3A7 (Fig. 4)^18^. The IC_50_ was determined to be 5.31 µM, which is similar to the *K*_M_ value of 5.4 µM^9^ for the 16α-hydroxylation of DHEA-S by CYP3A7. This suggested that DBF is a good surrogate substrate that likely binds in the CYP3A7 active site mirroring native substrates.

In order to test the proof-of-principal of utilizing DBF in a real world high-throughput screen of potential CYP3A7 substrates/inhibitors, we screened a mini-library of HCV and HIV antiviral drugs in order to assay their propensity for inhibition of DBF oxidation (Table 2, Fig. 3). HCV and HIV antiviral therapy occurs over long timeframes (for life, in the case of HIV) and the drug plasma levels must be maintained at high concentrations in order to adequately suppress viral replication. Additionally, the drugs are typically given as part of a drug cocktail that may contain multiple CYP substrates that are likely to act as inhibitors. These were the primary issues that undergirded our reasoning for selecting this type of mini drug library for our proof-of-principal test. It should be noted that, as seen in Table 2, several of these drugs have previously been reported to be CYP3A7 inhibitors^9,17,44^, while no data is available for the others. In this way, we were able to observe the effects on the assay of both a test set of “training” compounds and also “unknown” compounds. The data indicate that the most potent inhibitors were the HIV protease inhibitors, including: ritonavir, lopinavir, nelfinavir, darunavir, and amprenavir (Fig. 3), which have previously been reported to be inhibitors of CYP3A7 by us^9^ and others^17^. While the compound test set is necessarily small here, it indicates that the assay demonstrates robust performance against FDA approved drugs and is suitable to employ in a HTS format. Interestingly, danoprevir, an HCV protease inhibitor, dasabuvir, an HCV NS5B polymerase inhibitor, and glecaprevir, an HCV nonstructural (NS) protein 3/4A protease inhibitor, all of which had never been examined for their ability to inhibit CYP3A7 before, displayed pronounced inhibition (> 60%) of CYP3A7 in the assay, indicating the potential for DDI risk should these medications ever be used to treat the neonate in the future. Because several of the drugs tested in our CYP3A7 HTS assay have not previously been identified as CYP3A7 inhibitors (e.g., the HCV antivirals) and, therefore, there is no comparison to be made with these drugs to previous results, in order to further validate our assay we sought to select representative compounds where IC_50_ values for CYP3A7 inhibition had been previously reported. In light of this, we chose two well-known CYP3A inhibitors (ritonavir and lopinavir) where CYP3A7 inhibition data is available^9^ in order to determine if the previously reported values corresponded well with those retrieved from our HTS assay using DBF as the substrate. As demonstrated in Fig. 4, the IC_50_ obtained for ritonavir with CYP3A7 in the DBF assay were similar to those reported previously^9^. The slight discrepancy (< twofold) between the two sets of values is likely due to the difference in substrate affinities (with DBF being a higher affinity substrate than DHEA-S) and the analytical methods used (fluorescence plate reader assay vs. LC–MS). In any case, both assays demonstrate similar trends for each substrate and are within the biological margin of error, further validating the usefulness of the CYP3A7 HTS inhibition assay.

One potential concern with fluorogenic assays for CYP enzyme inhibition is the frequently weak correlation between data obtained with the fluorogenic assay and results obtained in LC/MS-based assays^45,46^. While LC/MS-based assays have a greater degree of sensitivity and accuracy, the equipment to perform these types of analytical experiments is relatively much more expensive and time-consuming to use when compared to fluorescent-based assays. Fluorescent-based assays, such as the one described herein, provide a robust and reliable way to detect the potential inhibitory effects of test compounds in a high-throughput fashion, which can then be followed up with LC/MS for validation. The assay described here also enables an efficient preliminary determination of intrinsic CYP3A7 inhibitory profiles by allowing for multi-point kinetic experiments to be conducted and determination of the *K*_i_ value of inhibitor compounds, as well as IC_50_, over a wider range of substrate concentrations.

Our approach provides a useful assay tool that can be used in concert with other high-throughput assays during the early stages of drug discovery and development. Using our approach, 45 compounds at one concentration can be tested in duplicate per 96-well plate. Future studies that we plan to undertake will include expanding this methodology to assess CYP3A7 activity in neonatal human liver microsomes. Judicious use of this assay to screen out drug candidates that may be potent CYP3A7 inhibitors will improve our ability to safely and effectively treat neonates, our most fragile patient population.

## Supplementary Information

Supplementary Information.

## Abbreviations

ACN: Acetonitrile
BFC: Benzyloxy-4-trifluoromethyl-coumarin
BOMCC: 7-Benzyloxymethyloxy-3-cyanocoumarin
CHC: 3-Cyano-7-hydroxycoumarin
CYP: Cytochrome P450
DBF: Dibenzylfluorescein
DBOMF: Dibenzyloxymethylfluorescein
DDI: Drug–drug interaction
DMF: Dimethylformamide
DMSO: Dimethylsulfoxide
HFC: 7-Hydroxy-4-trifluoromethyl-coumarin
HLMs: Human liver microsomes
HTS: High-throughput screening
LC–MS/MS: Liquid chromatography tandem mass spectrometry
NaOH: Sodium hydroxide
RBE: Resorufin benzyl ether
rCYP3A7: Recombinant CYP3A7
S/N: Signal-to-noise
7-BQ: 7-Benzyloxy-quinoline
7-HQ: 7-Hydroxyquinoline
