# Supplementary material for: Characterization of fluorescent probe substrates to develop an efficient high-throughput assay for neonatal hepatic CYP3A7 inhibition screening
Source: Sci Rep. 2021 Sep 30;11:19443. doi: 10.1038/s41598-021-98219-x (PMC8484451; doi:10.1038/s41598-021-98219-x)
Supplement: Supplementary file 1 — Supplementary Information. [file 41598_2021_98219_MOESM1_ESM.docx]

**Supplementary Information for Work, et al., “Characterization of fluorescent probe substrates to develop an efficient high-throughput assay for neonatal hepatic CYP3A7 inhibition screening”**

**Ref: Submission ID 5a74de6c-ba02-475c-8350-af90db434d6d**

**Supplemental Figures:**


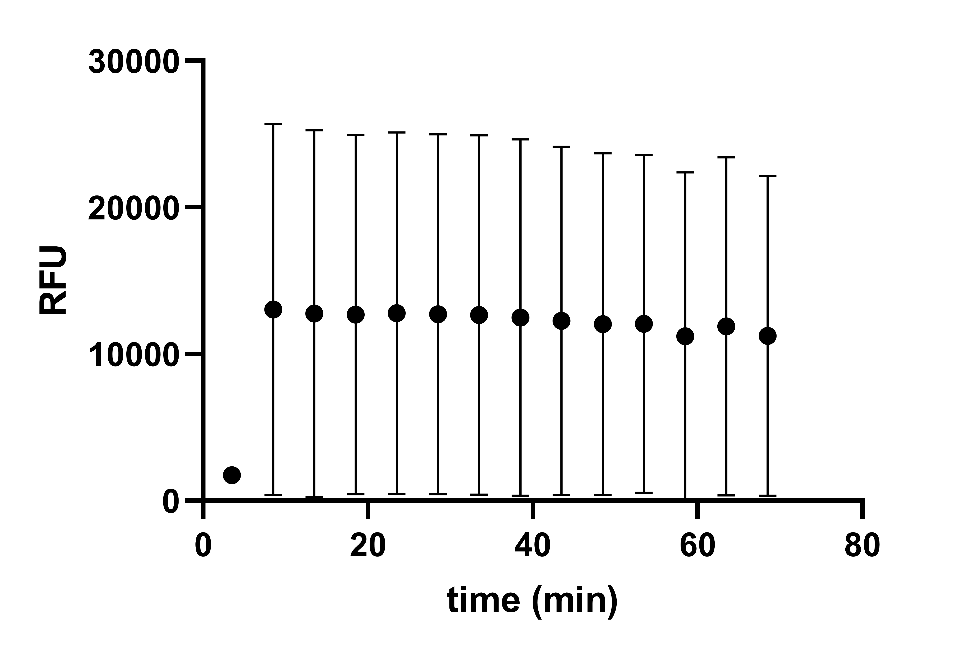


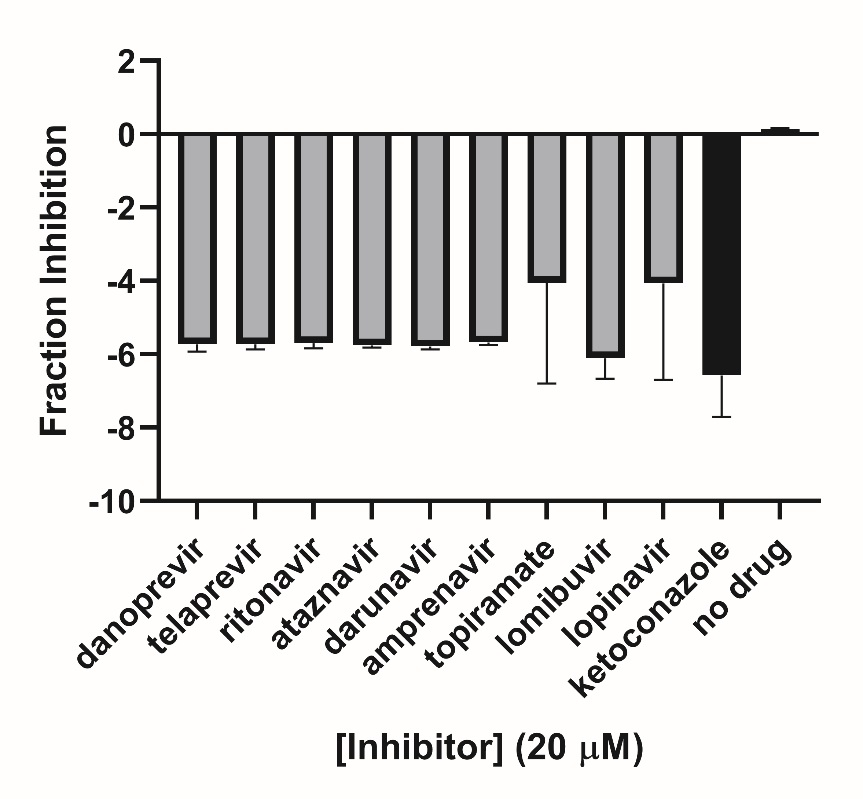
**Figure S1:** Nile Red kinetics by CYP3A7 enzyme. Each point represents the average of 3 replicates, and the error bars represent the standard deviation.

**Figure S2:** CYP3A7 inhibition by HCV and HIV inhibitors assessed using 7-BQ as a fluorescent marker. All compounds were tested in triplicate. Bars represent the average fraction of inhibition value, and the error bars represent the standard deviation. All values are negative due to the lack of difference between controls followed by subtraction of background signal.

**
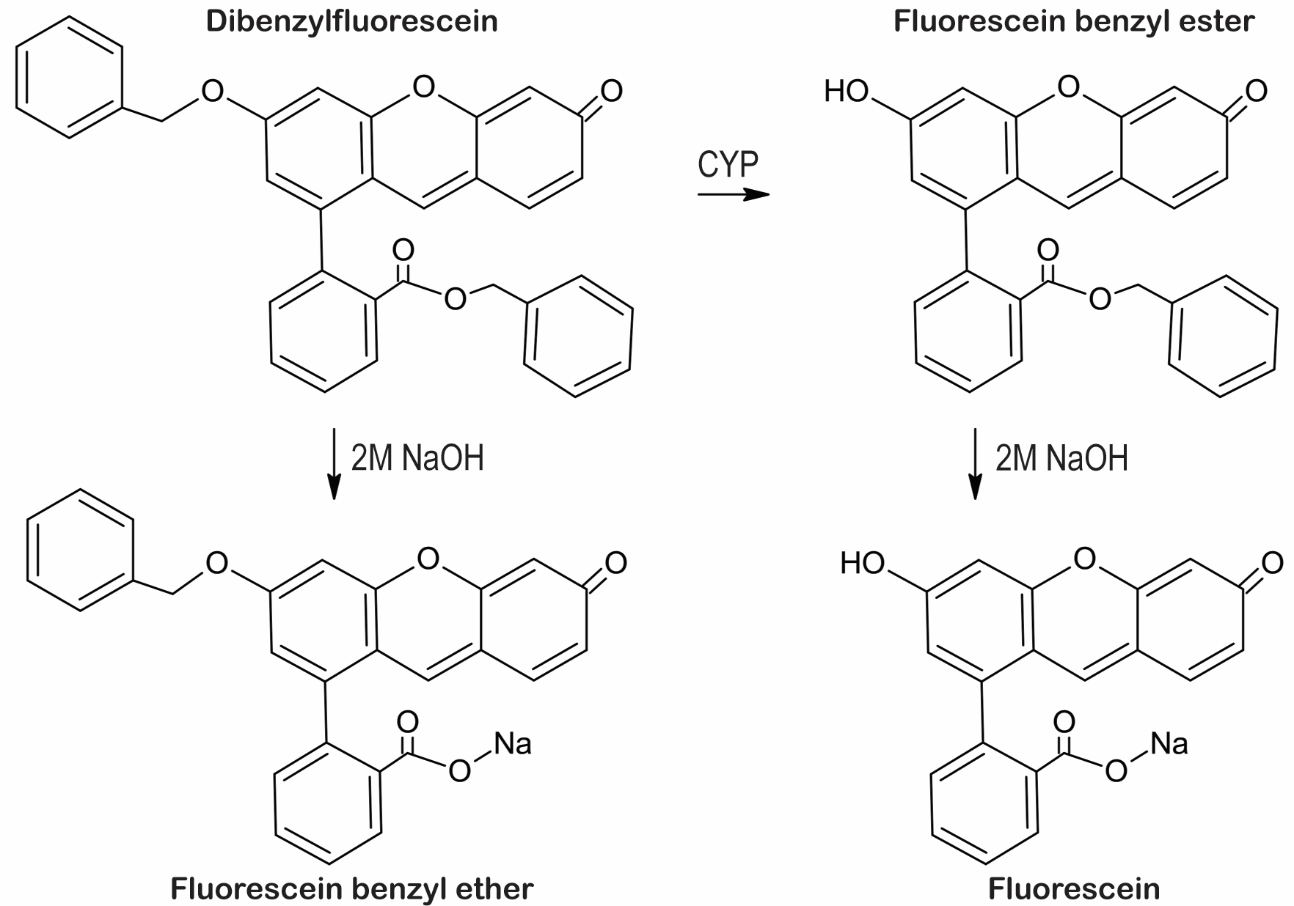
Figure S3:** Reaction schematic showing the structures of the probe substrate, dibenzylfluorescein (DBF), the *O*-dealkylated fluorescein benzyl ester (FBE) produced by CYP3A7, the fluorescein benzyl ether (FBEt), and fluorescent metabolic product fluorescein (F), produced after base-catalyzed ester hydrolysis.

**

Figure S4.** LC-MS confirmation of the fluorescein benzyl ester (FBE) metabolite formation by CYP3A7.

A. Representative total ion chromatograms (TIC) for DBF and its metabolites formed in CYP3A7 methanol-stopped incubation samples with and without NADPH and in no CYP3A7 control incubation sample. B. Representative MRM chromatograms of fluorescein metabolite (m/z 333.2>287.2) formed in DBF incubations with CYP3A7 in presence of NADPH and the effect of NaOH treatment. C. Representative MRM chromatogram of fluorescein benzyl ether metabolite (m/z 423.3>91.1) formed in the no NADPH control incubation after NaOH treatment, including representative MSMS spectrum of the base-hydrolyzed product. D. Representative MSMS spectra of the DBF standard, fluorescein formed in DBF incubation with CYP3A7 and after NaOH treatment, and fluorescein benzyl ether metabolite formed in DBF incubation with CYP3A7 and stopped with methanol. The MSMS spectrum of the fluorescein metabolite corresponds to the MSMS spectrum of the fluorescein standard. Fluorescein benzyl ether and ester MSMS spectra formed in DBF incubations present the characteristic fragment ions described in Salminen et al. (Ref 31).

**Materials and Methods for Supplemental Figure S4:**

*LC-MS Confirmation of Fluorescein Benzyl Ester Metabolite.* For metabolite characterization, DBF (10 µM) was incubated at 37 °C with 15 pmol/mL CYP3A7 Supersomes in 0.2 mL reactions containing 100 mM potassium phosphate buffer (pH 7.4) and 3 mM MgCl_2_. Reactions were initiated by the addition of the NADPH-regenerating system mix and stopped after 45 min with either 0.2 mL cold methanol or 0.15 mL 2 M NaOH. The methanol stopped samples were kept on ice and 0.15 mL of water was added. For the NaOH stopped samples, a second 45 min incubation at 37 °C was carried out and 0.2 mL cold methanol was then added. Control incubations without NADPH and without CYP enzyme were done in parallel. Precipitated proteins were collected by centrifugation of the stopped reaction samples for 20 min at 3,400 × g and 4 °C. Supernatants were transferred to high performance liquid chromatography (HPLC) vials and aliquots of 2 µL were analyzed by liquid chromatography tandem mass spectrometry (LC-MS/MS) with a Waters Acquity Ultra-Performance Liquid Chromatography (UPLC) system interfaced by electrospray ionization with a Waters Xevo TQ-S micro tandem quadrupole mass spectrometer (Waters Corp., Milford MA). Positive ionization mode with multiple reaction monitoring (MRM) or MSMS scan type were used for the metabolite characterization. The following source conditions were applied: 0.5 kV for the capillary voltage, 150 °C for the source temperature, 450 °C for the desolvation temperature, 50 L/h for the cone gas flow, 900 L/h for the desolvation gas flow and 25 V for cone voltage. Details regarding mass transitions, collision energies (CEs), and retention times are summarized for each respective analytes in Supplemental Table S1. DBF and its metabolites were separated on a Waters BEH C18 column (1.7 µm, 2.1 x 50 mm) by flowing 0.1 % formic acid in water and acetonitrile at 0.4 mL/min. The following gradient was used: 10% organic (acetonitrile) held for 0.5 min, increased to 98% over 4 min, and held at 98% for 0.9 min.

**Supplemental Table S1: LC-MS properties of DBF and its metabolites.**

| Analyte | Mass transition (*m/z*) | CE for MRM scan | Daughter scan of | CE for MSMS scan | Retention Time (min) |
| --- | --- | --- | --- | --- | --- |
| Dibenzylfluorescein (DBF) | 513.45>91.1  513.45>485.4 (only used for qualification) | 32  26 | 513.4 | 28 | 3.68 |
| Fluorescein | 333.2>287.2 | 32 | 333.2 | 40 | 2.51 |
| Fluorescein benzyl ester | 423.3>91.1 | 32 | 423.3 | 28 | 2.67 |
| Fluorescein benzyl ether |  |  |  |  | 3.61 |
